# Deliberate Self-Harm Among Patients Referring to the Emergency Room in Damghan, Iran

**Published:** 2014

**Authors:** Masuadeh Babakhanian, Maliheh Sadeghi, Elham Mohamadpur, Hanieh Rezazadeh

**Affiliations:** 1 Social Worker, Department of Social Work, Velayat Hospital, Semnan University of Medical Sciences, Damghan, Iran.; 2 Health Information Manager, Health Information Technology Group, School of Nursing and Paramedics, Semnan University of Medical Science, Semnan, Iran.; 3 Psychologist, Emam Khomeini Help Committee, Damghan, Iran.; 4 Family Therapist, University of Science & Culture, Tehran, Iran

**Keywords:** Comorbid Disorder, Deliberate Self-Harm, Prevalence, Substance Abuse

## Abstract

**Objective:**

Deliberate self-harm (DHS) is a deviant behavior that has been not completely emphasized in health-related studies in Iran. The current study was conducted to explore the prevalence and reasons associated with the incidence of DSH in patients referring to the emergency room of Baradaran-e Rezaee Hospital in Damghan, Iran.

**Methods:**

Fifty-four clients with the mean age of 29.4 (*±*10.3) years participated in this cross-sectional study in 2010. Firstly, demographics and details of substance use were collected based on items elicited from the Addiction Severity Index (ASI) and a researcher-made questionnaire. Then details of comorbidity and factors associated with the current DHS were collected by a semi-structured interview. Data were analyzed by performing descriptive methods of statistics.

**Results:**

Deliberate self-poisoning with using toxic substances such as lead, and poison (44.8%) followed by drug intoxication such as opiate and methamphetamine (35.2%) and body and vessel cutting (20%) were the most prevalent types of DSH. Furthermore, results revealed that comorbidities such as physical illness (38.9%) and psychiatric disorders including depression (31.5%), psychotic symptoms (15%), bipolar disorder (5.6%), stress (5.6%), and anxiety (1.9%) were commonly prevalent. The most frequently reported factors associated with DSH were desires for self-punishing (42%), self-medication for emotional sufferings (33%), experiencing euphoric feelings (24%), and stress (20%), respectively.

**Conclusion:**

DSH is one of the critical health and treatment priorities, which are prevalent in emergency rooms of hospitals in Iran. Clients with comorbid diagnosis of DSH, especially drug use warrant specific attention in emergency rooms.

## Introduction

Self-harm refers to a wide range of issues that individuals do to themselves in a deliberate way. Self-harm can consist of cutting, burning, banging or scratching ones, scalding, hair pulling, own body breaking bones, ingesting toxic objects, or substances. Scientific definition of deliberate self-harm (DSH) refers to the intentional, culturally unacceptable, self-performed, immediate, and direct destruction of the body. It is an act in which an individual deliberately initiates a non-habitual behavior that, without intervention from others, will cause lasting self-harm (1).

Self-harm has been placed in the Diagnostic and Statistical Manual of Mental Disorders, Fourth Edition Technical Revision (DSM-IV-TR) as a symptom of borderline personality disorder, but studies show that patients with other diagnoses may also do self-harm, including those with anxiety, depressive disorders, substance abusers, post-traumatic stress disorder, personality disorders, and even suicide (2).

The model of affect regulation suggests that DSH is utilized as a strong coping strategy to channel severe negative, painful, overwhelming, or extreme emotions into a physical modality presentation (3). Nixon et al suggest that DSH is associated with an inability to use regular symbols to self-report emotion, with self-harm substituted for verbal emotional expression (3). Many individuals who engage in DSH give emotional reasons for their behavior supporting this model of DSH. Hawton et al revealed in their study that this behavior can act as a serious mental health problem among young individuals and is associated with a wide range of maladaptive psychological and social problems (4).

Evidence suggests that DSH is becoming a global challenge in many countries of the world (5). Self-harm is a serious health problem with medical consequences, which can be associated with childhood sexual abuse, childhood physical abuse, emotional neglect, and problems with caregivers (6-8). 

Maladaptive psychological correlates of DSH behavior could be suicidal ideation, depression, anxiety, low self-esteem, and poor coping skills (9). Negative social experiences that are more common among individuals with a history of DSH and includes peer victimization and childhood abuse (10, 11). When DSH is no longer effective in managing emotional pain an individual may turn to suicide as a last resort (12).

The internal and external motivations for self-harm are diverse, and it may be used to fulfill different functions. These functions include self-harm being used as a coping strategy, which provides temporary relief of intense stressful feelings such as stress, depression, anxiety, and a sense of failure. Self-harm is often associated with a history of trauma and abuse, such as drug addiction. Studies show that there is also a strong relationship between self-harm and emotional abuse (13).

Aside from the immediate and potentially permanent physical damage that DSH can do the body, maladaptive and abnormal psychological contributors of DSH behavior and thought include poor coping skills, anxiety, depression, and low self-esteem are critical (14, 15). There are numerous long-term psychological and social consequences that deserve attention in the treatment of this problem because DSH can facilitate committing suicide (16).

There is evidence that in recent years, referrals of clients to emergency rooms in hospitals for DSH have become an increasing trend in Iran, especially because of using illicit drugs such as crystal methamphetamine. Although DSH is a common problem, but there is a paucity of research on this issue, especially in medical settings such as emergency rooms because this issue has important clinical and treatment implications for patients in hospitals. The current pilot study aims to preliminarily identify the incidence of DSHs among clients who had been referred for treatment to the emergency room of Baradaran-e Rezaee Hospital in Damghan City in and capital of Damghan County, Semnan Province of Iran, and also explores the factors associated with this health concern.

## Materials and Methods

The study design was descriptive cross-sectional. All study procedures were conducted in Baradaran-e Rezaee Hospital in Damghan, Iran during 2010. Fifty-four male and female patients with the mean age of 29.4 (±10.3) years were recruited through direct referrals of the physician of emergency rooms for study participation during that year. Inclusion criteria included age of 18-65 years; any current self-harm; current approved medical diagnosis of committing DSH by the physician of the emergency room; referring to the emergency room of Baradaran-e Rezaee Hospital for receiving inpatient or outpatient medical treatment services; and living in Damghan or in the suburbs at the onset of the study. Exclusion criteria included rejecting signing consent form. Participation was confidential.

All participants provided written informed consent after being fully informed of the potential benefits of participation. First, demographics including gender, age, education, marital status, living condition, and employment and details of illicit drug use were completed based on items elicited from the Addiction Severity Index (ASI) for those patients who had used illicit drugs such as opiates and a research-made questionnaire for those patients who used other toxic substances such as lead or had general DSHs such as vessel and body cuttings (17). The reliability and validity of ASI has been studied in previous studies in Iran (17). The reliability of the researcher-made questionnaire was approved by a 2-week test-pretest on 15 cases. Alpha Cronbach was 86% and face validity was approved by studying scientific local and international resources in this field and the questionnaire approval by researchers at Tehran University of Medical Sciences. Details of comorbidity were collected by the psychiatrist of the hospital. The factors associated with committing DSH were collected by semi-structured interviews for each participant. The general executive committee of the hospital approved the study procedure.

***Data analysis***

The study analysis was conducted by performing descriptive methods of statistics including percentage, mean, and standard deviation using SPSS for Windows (version 16.0, SPSS Inc., Chicago, IL, USA).

## Results

All clients were referred to the emergency room of Baradaran-e Rezaee Hospital for treatment during year 2010. The majority of clients (81.5%) were men while the remaining clients (18.5%) were female. Half of the sample had elementary education while the remaining clients had 12 years of education (31.5%) and more than 12 years of education (18.5%). Most of them reported that they were unmarried (55.6%) and employed (44.4%) at the time of referrals to the emergency room of Baradaran-e Rezaee Hospital. The majority of the clients (88.9%) were living in Damghan while the remaining clients (11.1%) were living in the suburbs of Damghan at the onset of study (see details of demographics in table 1).

Patients typically had self-poisoned with toxic substances such as lead and poison (44.8%), while illicit drug intoxication such as opiates was prevalent among some of them (35.2%) followed by the body and vessel cutting (20%). Among those cases that used illicit drugs and experienced severe intoxication, methamphetamine use, opiate use, and both opiate and methamphetamine consumption were prevalent at the time of referrals. The onset age of illicit drug initiation among the drug-using clients was 23.4 (±6.1) years and the length of illicit drug addiction was 3.5 (±3.7) years (see details of substance use in table 1).

Number of committing DSH was 2.3 (±1.5) times at the time of referral. Further statistical analysis revealed that comorbidity including physical illness (38.9%) and psychiatric disorders (59.6%) were commonly prevalent comorbidities among the sample (Table 2). 

**Table 1:** Demographic characteristics and details of substance use among the clients (n* = *54)

**Variable**	**Characteristics**	**%/SD** †
**Gender**	Male n (%)	44 (81.5)
	Female n (%)	10 (18.5)
**Age range (years)**		19-58
**Age (year)**		29 (±10.3)
**Education**	< 12 years n (%)	27 (50.0)
	12 years n (%)	17 (31.5)
	> 12 years n (%)	10 (18.5)
**Marital status**	Not currently married n (%)	30 (55.6)
	Currently married n (%)	24 (44.4)
**Living condition**	Damghan n (%)	48 (88.9)
	In the suburbs of Damghan n (%)	6 (11.1)
**Employment**	Employed n (%)	24 (44.4)
	Unemployed n (%)	23 (42.6)
	Student n (%)	7 (13.0)
**Type of substance of use at the time of deliberate self-harm**	Methamphetamine n (%)	23 (42.6)
	Opiate n (%)	16 (29.6)
	Opiate/methamphetamine n (%)	15 (2.8)
**Range of age in drug initiation (years)**		18-40
**Onset age of drug initiation (years)**		23.4 (±6.1)
**Length of drug addiction (years)**		3.5 (±3.7)

† Standard deviation

**Table 2:** Type of deliberate self-harm among the clients (n = 54)

**Variable**	**Characteristics**	**N (%/SD** † **)**
**Type of deliberate self-harm**	Self-poisoning with toxic substances such as lead n (%)	24 (44.8)
	Self-poisoning with illicit drugs such as opiates n (%)	19 (35.2)
	Body/vessel cutting n (%)	11 (20.0)
**Number of deliberate self-harm**		1-5
**Mean number of deliberate self-harm**		2 (±1.5)
**Comorbid disorders at the time of referral**	Physical illness n (%)	21 (47.7)
	Unipolar mood disorders n (%)	17 (31.5)
	Psychotic disorders n (%)	8 (15.0)
	Bipolar mood disorder n (%)	3 (5.6)
	Anxiety disorders n (%)	4 (7.5)
**Type of treatment received**	Inpatient treatment n (%)	38 (70.4)
	Outpatient treatment n (%)	16 (29.6)

† Standard deviation

The most frequently reported factors associated with DSH were desire for self-punishing (42%), self-medication for emotional sufferings (33%), experiencing euphoric feelings (24%), and stress (20%), respectively.

## Discussion

The current preliminary study is one the first studies that have emphasized the incidence of DSH and explored the factors associated with the incidence of this problem among referred cases to the emergency room of one hospital in the east of Iran.

One important finding in our study was the relatively young age of the clients at the time of referrals to the hospital and the prevalence of deliberate self-poisoning with highly toxic substances and illicit drugs. These issues are critical findings and need specific attention for medical management in emergency rooms of hospitals in the country. This study finding is consistent with a study showed that self-poisoning was prevalent, especially among young clients referring to emergency rooms of several hospitals (18).

The rate of DSH was higher in male clients in our study compared with female clients and was also frequently reported as a repeated deviant behavior. This study finding is consistent with a study showed that males are more engaged in DSHs than women (19, 4). Further studies with more representative samples of women are suggested.

The current study results showed that DSH was a prevalent health concern among the referred cases, especially among the referred drug abusers in this study. This study finding is consistent with other studies revealing that individuals who self-harm had higher rates of substance use disorders (7). This issue is important, so that emphasizes the increasing trend of presence of drug abusers in the emergency rooms of hospitals in Iran, which is a serious health concern for the healthcare system of the country.

One important new finding in our study, which has been less studied in our country, was the prevalence of DSH among psycho-stimulant drug users such as methamphetamine use, which is a new drug in Iran. This issue has important treatment and clinical implications for therapists and health policy makers in Iran and deserves extensive studies because the side-effects of methamphetamine is dramatically more problematic that traditional drug of Iran such as opium.

Prevalence of DSH with illicit drugs such as methamphetamine is a new emerging medical problem in our country that should be considered by physicians in referred cases to hospital emergency rooms because of detrimental effects that this highly potent and addictive drug would bring to the health condition of users and urgent needs for medical treatments in emergency rooms.

In addition, body and vessel cutting were also prevalent among participants of this study. In fact, body cutting and vessel cutting are the two prevalent types of DSH among individuals that referred for treatment in this study. This finding is consistent with other studies that emphasize the prevalence of body and vessel cutting among referred patients in emergency rooms of hospitals (8).

One important finding in our study was the prevalence of comorbid psychiatric disorders among the clients that were interviewed. Prevalence of DSH may be partly explained by the frequency of these disorders among our participants though it is subject to further study and cannot be explained with the current study with a small sample size. This is in line with many studies revealing that self-harm is often associated with other psychological problems and it tends to be treated under the umbrella of a co-occurring disorder like a substance abuse problem. Sometimes the underlying feelings that cause the self-harm are the same as those that cause the co-occurring disorder (8).

Participants frequently reported that the desire to self-punishing was an important reason with committing DSH while self-medication for emotional sufferings, experiencing euphoric feelings and relieving stress were the other factors. These findings may partly explain that our clients tried to use emotional coping strategies to separate themselves from comorbidity rather than logical and rational strategies of problem solving. This finding confirms a study, which showed that emotional problems are the major risk factors for DSH among patients who committed that (19). However, this issue is subject to further studies and is an avenue for further researches with bigger samples of referred substance abusers.

To sum up, hospital admission for DSH is a serious health concern. There are several limitations to the current study, including methodology, which was based on patients’ self-report and the cross-sectional design of study, and because of the small sample size; thus, we cannot generalize the findings of this study to other hospitals and cities in Iran.

One of the main study limitations was the lack of assessing personality disorders among the sample. Further studies on this issue are suggested.

We should note that it is an introductory study on the prevalence of DSH among referred cases to the emergency room of a hospital in Damghan, so further studies are still suggested.

Nevertheless, our findings are subject of attention to clinicians and therapists; further treatment should be conducted in hospitals to ensure that sufficient attention is being paid to the detection and management of DSH attempters with substance abuse problem, especially users of new drugs such as methamphetamine. Focusing on psychological functioning and self-esteem may be especially important among the referred clients, while working through environmental issues, which could contribute to committing DSH may also be especially important. For highly at risk DSH clients, perhaps, pharmaceutical treatment, followed by dialectical behavior therapy (20) is suggested.

Although, a recent Iranian study emphasized the high frequency of emergency psychiatric visits in an Emergency Department of Rasoul-e-Akam Hospital, Tehran, Iran (21), but further studies are still required to conduct on DSH related visits among different categories of substance users in emergency rooms of hospitals in the country. 

## Notes

**Declaration of interest:** None.
